# A Population-Based Study of Genetic Variation and Psychotic Experiences in Adolescents

**DOI:** 10.1093/schbul/sbt146

**Published:** 2013-10-30

**Authors:** Stanley Zammit, Marian Hamshere, Sarah Dwyer, Lyudmila Georgiva, Nic Timpson, Valentina Moskvina, Alexander Richards, David M Evans, Glyn Lewis, Peter Jones, Michael J. Owen, Michael C. O’Donovan

**Affiliations:** ^1^Institute of Psychological Medicine and Clinical Neurosciences, MRC Centre for Neuropsychiatric Genetics and Genomics, Cardiff University, Cardiff, UK;; ^2^Centre for Academic Mental Health, School of Social and Community Medicine, University of Bristol, Bristol, UK;; ^3^Department of Psychiatry, University of Cambridge, Cambridge, UK

**Keywords:** psychosis, schizophrenia, epidemiology, ALSPAC, GWAS, polygenic

## Abstract

Psychotic experiences are not uncommon in general population samples, but no studies have examined to what extent confirmed risk variants for schizophrenia are associated with such experiences. A total of 3483 children in a birth cohort study participated in semistructured interviews for psychotic experiences at ages 12 and 18. We examined whether (1) a composite measure of risk for schizophrenia conferred by common alleles (polygenic score) was associated with psychotic experiences, (2) variants with genome-wide evidence for association with schizophrenia were associated with psychotic experiences, and (3) we could identify genetic variants for psychotic experiences using a genome-wide association (GWA) approach. We found no evidence that a schizophrenia polygenic score, or variants showing genome-wide evidence of association with schizophrenia, were associated with adolescent psychotic experiences within the general population. In fact, individuals who had a higher number of risk alleles for genome-wide hits for schizophrenia showed a decreased risk of psychotic experiences. In the GWA study, no variants showed GWA for psychotic experiences, and there was no evidence that the strongest hits (*P* < 5 × 10^−5^) were enriched for variants associated with schizophrenia in large consortia. Although polygenic scores are weak tools for prediction of schizophrenia, they show strong evidence of association with this disorder. Our findings, however, lend little support to the hypothesis that psychotic experiences in population-based samples of adolescents share a comparable genetic architecture to schizophrenia, or that utilizing a broader and more common phenotype of psychotic experiences will be an efficient approach to increase understanding of the genetic etiology of schizophrenia.

## Introduction

The public health burden caused by schizophrenia and other psychotic disorders is a strong driver for research aimed at understanding the biological mechanisms underlying etiology that might potentially impact upon development of novel treatments. Although schizophrenia is an uncommon illness characterized by a relatively diverse phenotype, psychotic experiences are a key feature of this disorder and are reported by approximately 5%–10% of the general population.^1^ Such experiences are associated with an increased risk of developing psychotic disorders later in life.^2–7^ This has driven a body of research using psychotic experiences as a more common and potentially more powerful alternative phenotype to the clinical disorder itself, the premise being that psychotic experiences offer a strong indicator of underlying risk, which is only sometimes fully expressed as a clinical disorder. However, the positive predictive value of psychotic experiences in these studies is low, and therefore it is not clear to what extent studying this phenotype will aid understanding of the pathogenesis of schizophrenia, including its genetic etiology.

Recent findings from genome-wide association studies (GWAS) and genome-wide studies of copy number variation provide strong evidence, implicating a number of genetic loci as risk factors for schizophrenia.^8^ There is also increasing evidence of shared genetic contribution across different psychotic disorders, with most research based on GWAS data focusing on the overlap between schizophrenia and bipolar disorder.^8–10^ For example, a composite measure of risk conferred by common alleles (known as a polygenic score) derived from a discovery schizophrenia sample has been shown to successfully discriminate individuals with schizophrenia, and with bipolar disorder, in other samples.^11^

Twin studies provide some support that psychotic experiences are also heritable,^12,13^ but there has been a lack of studies examining the impact of specific genetic loci on psychotic experiences,^14^ and no studies have examined to what extent confirmed risk variants for schizophrenia are associated with such experiences in a general population sample. The extent to which the genetic architecture of psychotic experiences in the general population is comparable to that of schizophrenia is, therefore, unknown.

Our primary aim in this study was to examine whether a composite measure of risk for schizophrenia, conferred by common alleles and derived using the largest published GWAS of this disorder, shows association with psychotic experiences. As secondary aims we also examine:

• Whether specific variants within a number of putative risk genes for schizophrenia, selected on the basis of (1) GWAS findings for schizophrenia and (2) positional or functional candidates for this disorder, are associated with psychotic experiences
• Whether we could identify genetic variants for psychotic experiences during adolescence using a GWA approach, and to what extent these show overlap with genetic findings for schizophrenia

## Methods

### Sample

This study examines data from 3483 children from the Avon Longitudinal Study of Parents and Children (ALSPAC) cohort who (1) participated in the Psychosis-Like Symptoms interviews (PLIKSi) when they were aged 12 (*N* = 6796) and 18 years (*N* = 4724) and (2) had genetic data available (analysis restricted to 1 child per nuclear family). The initial cohort consisted of 14 062 children born to residents of the former Avon Health Authority area who had an expected date of delivery between April 1, 1991 and December 31, 1992 (www.alspac.bris.ac.uk). The cohort was set up to examine genetic and environmental determinants of health and development (see Fraser et al^15^ and Boyd et al^16^ for further details). All subjects and their parents gave written informed consent for this study. Ethical approval for the study was obtained from the ALSPAC Law and Ethics Committee and the Local Research Ethics Committees.

### Measures

#### Psychotic Experiences. Psychotic experiences

were assessed at ages 12 and 18 years using the semistructured PLIKSi,^7,17^ which draws on principles of standardized clinical examination developed for the Schedule for Clinical Assessment in Psychiatry (SCAN). It includes 11 “core” questions eliciting key psychotic experiences, covering hallucinations (visual and auditory), delusions (spied on, persecution, thoughts read, reference, control, grandiosity), and experiences of thought interference (broadcasting, insertion and withdrawal). Any unspecified delusions elicited were also rated. Cross-questioning was used to establish presence of symptoms, and coding followed glossary definitions and rating rules for SCAN.

Interviewers were psychology graduates trained in assessing the SCAN Psychosis Section and using the PLIKSi, and rated experiences as not present, suspected, or definitely psychotic. Unclear responses after probing were always “rated down,” and symptoms only rated as definite when a clear example was provided. The average κ values for interrater reliability at ages 12 and 18 were 0.72^17^ and 0.83,^7^ respectively.

Our primary outcome was an interviewer rating of one or more definite psychotic experiences at either age 12 or age 18, compared to a rating of none at both time points. As a secondary analysis, we also examined associations with a broader outcome of any suspected or definite experiences compared with none at these time points.

#### Polygenic Score.

We used the polygenic score analytic approach^18^ using the Schizophrenia Psychiatric Genome-Wide Association Study Consortium (PGC-SCZ) data set^19^ as a discovery sample to identify alleles with which to generate polygenic scores in the test (ALSPAC) subjects. Following the approach of the International Schizophrenia Consortium (ISC),^11^ sets of single nucleotide polymorphisms (SNPs) were selected at a range of values (*P* < .5, *P* < .4, *P* < .3, *P* < .2, *P* < .1, *P* < .05, and *P* < .01) for evidence of association with schizophrenia in the PGC-SCZ sample and were used to derive scores in the ALSPAC sample (from 101 200 SNPs following linkage disequilibrium [LD] pruning using a sliding window of 200 SNPs, at 5 SNP intervals, with an *r* ^2^ cutoff value of .2). The derived scores were based on the average number of risk alleles, each weighted by the effect size (lnOR) at each locus within the PGC-SCZ.

#### Single SNP Associations.

SNPs were divided into 2 categories depending upon the strength of evidence for their association with schizophrenia.

##### Category 1 

SNPs were selected for inclusion if they were reported as showing genome-wide levels of significance (*P* < 5 × 10^−8^) for association with schizophrenia, or a combined schizophrenia bipolar phenotype, and were not in strong LD (measured as *r* ^2^) with other included SNPs (where *r* ^2^ ≥.2, the most significant SNP from the PGC-SCZ was chosen). A search of studies in samples of European ancestry up to December 31, 2011 identified 17 SNPs (13 within different genes and 4 in intergenic segments) that met these criteria. Ten SNPs were selected from the PGC-SCZ sample^19^ (*MIR137* rs1625579, *ITIH3*/*4* rs2239547, *TRIM26* rs2021722, *CSMD1* rs10503253, *CNNM2* rs7914558, *NT5C2* rs11191580, *CACNA1C* rs4765905 and intergenic SNPs rs17662626 [343kb from *PCGEM1*], rs7004633 [421kb from *MMP16*], and rs12966547 [126kb from *CCDC68*]). Two of these (*ITIH3*/*4* rs2239547 and *CACNA1C* rs4765905) originally showed genome-wide significance for schizophrenia and bipolar samples combined but have shown genome-wide evidence for schizophrenia since.^10^ Six SNPs selected showed genome-wide evidence of association in meta-analyses of the ISC, S-GENE, and Molecular Genetics of Schizophrenia (MGS) samples (*NOTCH4* rs3131296, *PRSS16* rs6932590, *NRGN* rs12807809, *TCF4* rs9960767),^11,20,21^ or extended meta-analyses with other samples (rs2312147, 50 kb from *VRK2* ^22^ and *ZNF804A* rs1344706^23,24^), and 1 SNP was selected from a consortium of Western European samples (*AMBRA1* rs11819869).^25^

As an overall test of the hypothesis that these 17 SNPs that show the strongest evidence of association with schizophrenia are also associated with psychotic experiences, we constructed a risk score whereby a score of 1 was given for the presence of each risk allele at each independent SNP (*r* ^2^ < .2), and then scores summed across SNPs to obtain an individual risk score. We then tested for association between the risk score (using both unweighted scores and scores weighted by effect sizes in the discovery samples) and our outcome measures.

##### Category 2 

We examined a number of SNPs within genes (including *PTBP2*, *NRXN1*, *FXR1*, *HLA*-*DQA1*, *MAD1L1*, *ANK3*, *NOS1*, and *RPGRIP1L*) or intergenic regions that showed suggestive (*P* < 5 × 10^−5^), but not genome-wide, evidence of association in GWAS. We also examined SNPs within a number of genes (most identified through positional approaches) either hypothesized to play a functional role on the pathogenesis of schizophrenia through affects on neurotransmitter metabolism (*COMT*, *DAOA*), receptor function (*DRD2*, *GRM3*, *CHRNA3*), myelination (*OLIG2*, *MAGI2*, *PTPRZ1*, *QKI*, *CNP*, *NRG1*, *ERBB4*), synaptic function (*DTNBP1*), or microtubule function (*DISC1*), or positional candidates (eg, *TBX1* and *GNB1L* within the 22q11 deletion syndrome).

#### Genome-Wide Association Studies.

A total of 9912 ALSPAC children were genotyped using the Illumina HumanHap550 quad genome-wide SNP genotyping platform by 23andMe subcontracting the Wellcome Trust Sanger Institute, Cambridge, UK and the Laboratory Corporation of America, Burlington, NC. Individuals were excluded from further analysis on the basis of having incorrect gender assignments, minimal or excessive heterozygosity (<0.320 and >0.345 for the Sanger data and <0.310 and >0.330 for the LabCorp data), disproportionate levels of individual missingness (>3%), evidence of cryptic relatedness (>10% of alleles identical by descent), and being of non-European ancestry (as detected by a multidimensional scaling analysis seeded with HapMap 2 individuals. EIGENSTRAT analysis revealed no additional obvious population stratification, and genome-wide analyses with other phenotypes indicate a low genomic inflation factor, with λ ≈ 1. The resulting data set consisted of 8365 individuals and 500 527 SNPs. SNPs with a minor allele frequency of <1% and call rate of <95% were removed. Furthermore, only SNPs, which passed an exact test of Hardy–Weinberg equilibrium (*P* >5×10^–7^), were considered for further use. Genotypes were imputed with MACH 1.0.16 Markov Chain Haplotyping software,^26,27^ using CEPH individuals from phase 2 of the HapMap project as a reference set (release 22).

### Statistical Analysis

Logistic regression was used to test for association between each SNP and our psychosis outcomes under an additive genetic model. Results are presented as ORs and 95% CIs per copy of test allele (see table 2) for individual SNPs, per schizophrenia risk allele increase for risk scores, and per SD for polygenic scores. Nonlinear associations between polygenic scores and outcomes were examined by inclusion of quadratic terms in the regression models. Adjusting for sex made no difference to any of the estimates reported. A binomial test was used to examine whether SNPs showing evidence of association with psychotic experiences in the GWAS showed greater than chance evidence of association with schizophrenia in the PGC-SCZ sample. Analyses were conducted using Stata (version 11), PLINK (version 1.06),^28,29^ and mach2dat.^26,27^

## Results

There were 4060 individuals who participated in the PLIKSi at ages 12 and 18 years, and 3483 (85.8%) of these had genetic data available that passed strict quality control criteria. Of these, 424 (12.2%) had definite psychotic experiences at either age.

### Schizophrenia Polygenic Score

There was no evidence that individuals who had higher polygenic scores (reflecting increased genetic risk for schizophrenia) were at an increased risk of definite psychotic experiences during adolescence (table 1). Although, on average, individuals with definite psychotic experiences had higher polygenic scores than those without, there was, at best, only very weak evidence (without any correction for multiple testing) that this was different from random variation (strongest evidence at discovery sample *P* value cutoff <.3; OR per SD increases in score = 1.08, 95% CI: 0.98, 1.20; *P* = .134). There was no evidence of any nonlinear association with polygenic scores (*P* values for quadratic terms ranged from .5 to .9; see online supplementary table S1).

**Table 1.** Association Between Schizophrenia Polygenic Score (Per SD^a^) and Definite Psychotic Experiences at Age 12 or 18

*P* Value Cutoff in Discovery Sample	OR	LCI	UCI	*P*	Same Direction as Discovery Sample
.5	1.08	0.97	1.19	.158	+
.4	1.06	0.96	1.18	.247	+
.3	1.08	0.98	1.20	.134	+
.2	1.05	0.95	1.17	.311	+
.1	1.06	0.95	1.17	.296	+
.05	1.07	0.96	1.18	.230	+
.01	1.08	0.97	1.20	.148	+

*Note*: LCI, L95% CI; UCI, U95% CI.

^a^No evidence of nonlinearity (including quadratic terms).

### Association With Schizophrenia Genome-Wide Significant SNPs

Three of the 17 schizophrenia genome-wide significant SNPs tested (table 2) showed some evidence of association with definite psychotic experiences (*MIR137* rs1625579 on chromosome 1, *P* = .012; intergenic SNP rs17662626 near *PCGEM1* on chromosome 2, *P* = .017; and *NT5C2* rs11191580 on chromosome 10, *P* = .036). However, for each of these SNPs, the risk allele for schizophrenia was *reduced* in frequency in those with definite psychotic experiences.

**Table 2.** Association Between Schizophrenia Genome-Wide Significant SNPs and Definite Psychotic Experiences at Age 12 or 18

Ch.	Gene	SNP	A1^a^	A2^a^	Frequency (A1)	OR^b^	L95% CI	U95% CI	*P*	As SCZ^c^
1	*MIR137*	rs1625579	G	*T*	0.18	1.25	1.05	1.49	.012	−
1	*VRK2* (IG)	rs2312147	*C*	T	0.62	0.99	0.85	1.15	.897	−
2	*ZNF804A*	rs1344706	*A*	C	0.59	1.05	0.90	1.21	.536	+
2	*PCGEM1* (IG)	rs17662626	*A*	G	0.92	0.75	0.59	0.95	.017	−
3	*ITIH3*/*4*	rs2239547	C	*T*	0.27	1.05	0.89	1.24	.550	−
6	*PRSS16*	rs6932590	C	*T*	0.25	0.99	0.84	1.18	.935	+
6	*TRIM26*	rs2021722	*C*	T	0.77	1.10	0.93	1.31	.274	+
6	*NOTCH4*	rs3131296	*C*	T	0.85	0.96	0.79	1.18	.717	−
8	*CSMD1*	rs10503253	*A*	C	0.19	0.97	0.81	1.16	.729	−
8	*MMP16* (IG)	rs7004633	A	*G*	0.84	1.19	0.96	1.47	.107	−
10	*CNNM2*	rs7914558	A	*G*	0.39	1.06	0.92	1.23	.425	−
10	*NT5C2*	rs11191580	C	*T*	0.08	1.31	1.02	1.69	.036	−
11	*NRGN*	rs12807809	C	*T*	0.17	1.07	0.89	1.29	.478	−
11	*AMBRA1*	rs11819869	*C*	*T*	0.82	0.89	0.74	1.07	.204	+
12	*CACNA1C*	rs4765905	*C*	G	0.34	0.97	0.83	1.13	.668	−
18	*CCDC68* (IG)	rs12966547	A	*G*	0.40	1.13	0.97	1.31	.109	−
18	*TCF4*	rs9960767	A	*C*	0.95	1.03	0.73	1.44	.881	−
Risk score (17 genome-wide hits)						0.96^d^	0.92	0.99	.024	−

*Note*: SNP, single nucleotide polymorphism; ch., chromosome; IG, intergenic; A1/A2, allele 1/allele 2, L95% CI, lower 95% CI; U95% CI, upper 95% CI; PGC-SCZ, Schizophrenia Psychiatric Genome-Wide Association Study Consortium.

^a^Schizophrenia risk alleles are italicized.

^b^Per allele 1.

^c^Risk allele for psychotic experiences same as for schizophrenia (SCZ) in PGC-SCZ sample (+), or different (−).

^d^Per risk allele (unweighted).

There was evidence of association between the risk score derived from the 17 schizophrenia genome-wide significant SNPs and definite psychotic experiences (OR per 1-point score increase = 0.96, 95% CI: 0.92, 0.99; *P* = .024), though again this indicated that psychotic experiences were less likely in those with more schizophrenia risk alleles.

### Association With Other Schizophrenia Candidate SNPs

Of 61 SNPs (47 SNPs at *r* ^2^ <.2) examined (online supplementary table S2), 6 showed some evidence of association with definite psychotic experiences (*NRXN1* rs3850333, *ERBB4* rs4673628, *GRM3* rs6465084, *MAGI2* rs6951046, *GNB1L* rs2269726, and *COMT* rs2097603), but none of these associations persisted after correction for multiple testing.

### GWAS of Psychotic Experiences

**Fig. 1.**
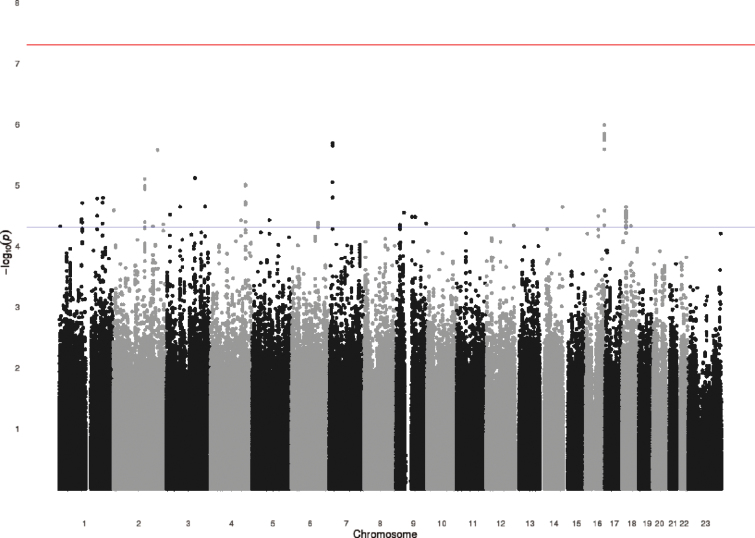
Manhattan plot for the genome-wide association studies of definite psychotic experiences at ages 12 or 18. The Y-axis shows –Log (*P* values) of the logistic regression analysis (additive genetic model) for single nucleotide polymorphisms tested with definite psychotic experiences at ages 12 or 18 compared to none at both time points.

**Fig. 2.**
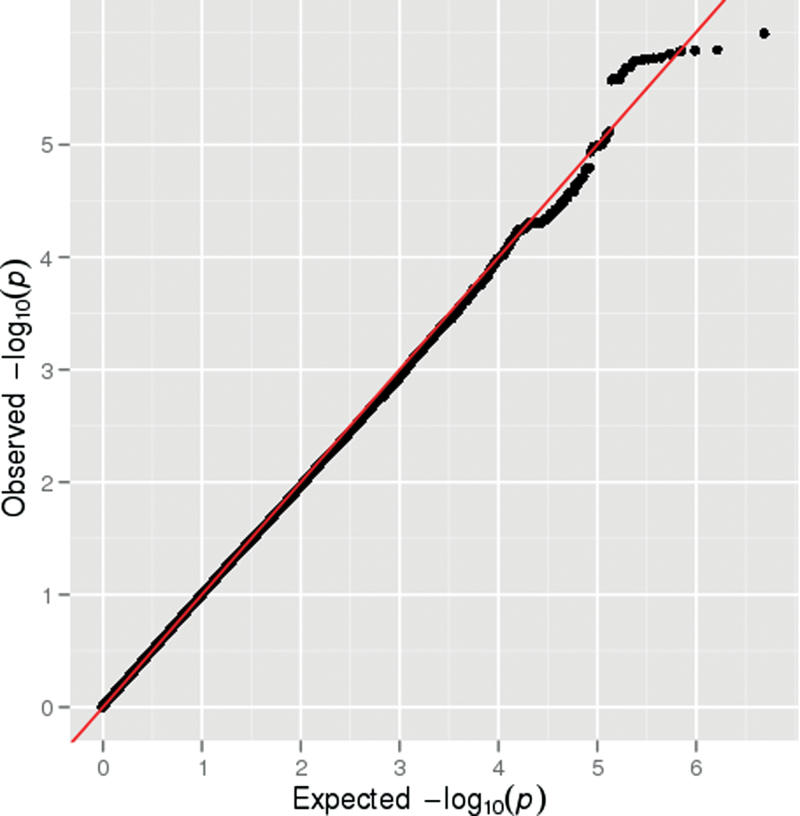
Q-Q plot for the genome-wide association studies of definite psychotic experiences at ages 12 or 18.

Results of the GWAS are shown in figure 1. A quantile-quantile (Q-Q) plot of the association test *P* values did not show deviation from what was expected under the assumption of no genetic association (figure 2), and the value of λ was 1.0. None of the 2 487 019 SNPs tested showed genome-wide evidence (*P* < 5 × 10^−8^) for association with definite psychotic experiences. There were 121 SNPs that showed moderate evidence of association (*P* < 5 × 10^−5^), with 31 SNPs representing probable independent signals (*r* ^2^ < .2) (table 3; figure 1). There was no evidence that these suggestive hits for psychotic experiences were enriched for genetic variants associated with schizophrenia in the PGC-SCZ sample (binomial test, *P* > .99).

**Table 3.** Genome-Wide Association Study^a^ of Psychotic Experiences at Age 12 or 18 vs None

Ch.	BP	Gene	SNP	ALSPAC						PGC-SCZ
				Allele	Frequency 1	*P*	OR	SE	*P*	*P*
1	4677487	*AJAP1*	rs16839502	A,T	0.9569	.7327	0.507	0.167	4.79E-05	.085
1	102105577	*OLFM3*	rs17125624	C,G	0.8685	.9969	0.657	0.102	3.65E-05	.386
1	105942799	Null	rs4489613	G,T	0.8312	.9562	1.629	0.114	1.99E-05	.137
1	173693700	*TNR*	rs863516	A,G	0.0355	.4617	2.834	0.242	1.68E-05	.225
1	198349578	*NR5A2*	rs2249172	A,G	0.4802	.8229	1.417	0.082	1.96E-05	.935
1	200012862	*NAV1*	rs523218	G,T	0.9374	.9967	0.556	0.136	1.63E-05	.110
2	2752976	Null	rs11127336	C,T	0.9809	.6373	0.328	0.265	2.62E-05	.218
2	143889170	*ARHGAP15*	rs2381435	A,T	0.1918	.9965	1.48	0.088	8.02E-06	.105
2	180239734	*ZNF385B*	rs17770666	C,T	0.1476	.9488	0.602	0.125	4.78E-05	.636
2	202453883	*PFTK2*	rs2192879	A,G	0.9179	.9962	0.581	0.116	2.67E-06	.451
2	227777254	*COL4A3*	rs6756117	A,G	0.6206	.9982	0.739	0.074	4.45E-05	.061
3	16065924	Null	rs987858	A,T	0.2843	.4475	0.579	0.131	3.09E-05	.043
3	61605054	*PTPRG*	rs12489977	C,T	0.6098	.9978	0.727	0.075	2.29E-05	1.000
3	130025938	Null	rs13082400	A,G	0.9751	.8266	0.398	0.206	7.72E-06	.848
3	176258264	*NAALADL2*	rs13319550	A,G	0.9418	.9631	0.546	0.143	2.27E-05	.822
4	140771849	Null	rs1027472	C,T	0.4784	.9984	1.362	0.075	3.82E-05	.525
4	160707756	Null	rs9992703	G,T	0.5924	.9993	1.416	0.079	9.92E-06	.300
5	78903305	Null	rs6453460	A,T	0.1714	.9088	1.507	0.099	3.79E-05	.507
6	119566383	*MAN1A1*	rs9489621	C,T	0.5864	.9981	1.376	0.078	4.16E-05	.232
7	15192064	Null	rs6962150	G,T	0.8287	.9957	0.647	0.092	2.06E-06	.710
9	17521968	Null	rs2593371	C,T	0.7013	.9451	0.718	0.081	4.85E-05	.677
9	36304566	Null	rs17385743	C,T	0.8188	.9975	0.693	0.088	2.86E-05	.585
9	71588335	Null	rs11521712	A,G	0.8995	.9982	0.632	0.111	3.36E-05	.381
9	87043180	Null	rs7040848	A,G	0.5564	.9969	0.734	0.075	3.37E-05	.937
9	136727589	*COL5A1*	rs4841924	A,G	0.9536	.6804	0.48	0.179	4.32E-05	.690
12	126484085	Null	rs1879390	A,C	0.1164	.966	1.551	0.108	4.64E-05	.837
14	102183305	*RCOR1*	rs714827	A,G	0.9154	.9958	2.16	0.182	2.32E-05	.943
16	59019380	Null	rs11643723	A,G	0.0489	.9951	1.857	0.149	3.26E-05	.532
16	86171466	Null	rs17781622	A,G	0.8951	.9319	0.578	0.112	1.03E-06	.903
18	17892308	Null	rs8086768	A,T	0.0942	.9964	1.613	0.113	2.31E-05	.323
18	40546336	*SETBP1*	rs2852779	G,T	0.1542	.9994	1.471	0.095	4.71E-05	.187

*Note*: ALSPAC, Avon Longitudinal Study of Parents and Children; BP, base-pair position. Abbreviations are explained in the first footnote to table 2.

^a^All independent (*r* ^2^ < .2) SNPs with evidence of association at *P* <5 × 10^−5^.

### Secondary Analyses

Results for polygenic score and candidate SNPs were similar when we examined a broader outcome of suspected or definite psychotic experiences (*N* = 912 [26.1%]) compared to none (2588 [73.9%]) (online supplementary tables S3–S5). No SNPs showed genome-wide evidence of association with this broader outcome, though 106 SNPs showed suggestive evidence of association, with 36 SNPs representing probable independent signals. There was no evidence that these suggestive hits were enriched for genetic variants associated with schizophrenia (binomial test, *P* = .297).

## Discussion

We utilized a number of approaches to examine the extent to which genetic risk factors for schizophrenia are shared with those for psychotic experiences within the general population. We found no evidence that a schizophrenia polygenic score was associated with adolescent psychotic experiences, or that genetic variants, showing genome-wide evidence of association with schizophrenia, were associated with psychotic experiences within the general population. In fact, we observed some evidence contrary to that hypothesized, with individuals who had a higher number of risk alleles for genome-wide hits for schizophrenia showing a decreased risk of psychotic experiences in our sample.

Although a potential explanation for the lack of association between the schizophrenia polygenic score and psychotic experiences in our sample is low statistical power (eg, see Dudbridge^30^ for a discussion of power in relation to the variance in liability explained by polygenic score, given model assumptions), this seems an unlikely explanation for our findings. Further examination of data from the PGC-SCZ sample reveals that the odds of schizophrenia within individual studies are increased approximately 1.5-fold (lowest CI: approximately 1.3) for each SD increase in polygenic risk score derived excluding the target study. Based on this empirical data, our study has >99% power to detect an effect of this magnitude. Furthermore, from our results, we can be highly confident of excluding an effect size greater than 1.2 per SD increase in score.

It would be surprising if genetic factors associated with schizophrenia risk do not have any influence at all on incidence of psychotic experiences in the general population. However, as our results demonstrate, if polygenic risk for schizophrenia increases risk for psychotic experiences, its impact on the latter is very much weaker than for the clinical disorder. This is contrary to expectations if the presence of psychotic experiences offers a strong indicator of underlying genetic risk. General population studies with larger samples or which utilize more accurately defined polygenic risk for schizophrenia will be required to provide evidence of association for such weaker effects.

Even if such evidence is forthcoming however, such a polygenic score is likely to be a weak tool for prediction of risk for psychotic experiences.^30,31^ For example, in the MGS and Cardiff samples that were used to validate the polygenic score approach for schizophrenia, the polygenic score explained <5% of the variance for this disorder even though there was very strong statistical evidence of association between a polygenic score and schizophrenia in these samples.^11^ The relatively weak predictive power of the polygenic score for schizophrenia may have limited the ability to observe an association with psychotic experiences in our study.

Samples of individuals with schizophrenia are likely to be enriched for genetic variants related to onset, severity, and chronicity of this disorder, while the effect of common schizophrenia risk alleles (the sort mainly accessed by polygenic score) may be on the development of cognitive abnormalities, negative symptoms, or thought disorder rather than on development of delusions or hallucinations, which were the only aspects of the schizophrenia phenotype measured in this study. A recent case-control study of schizophrenia found no evidence of association between a polygenic score and positive or negative dimensions of psychosis in controls, although this was a sample of only 148 individuals.^32^ It is also possible that the genetic contribution to the development of psychotic experiences changes over the life course, similar to depression and IQ^33^ and is relatively small during adolescence.

The finding that 3 of the 17 SNPs, showing genome-wide evidence of association of schizophrenia, showed association between the “protective” allele for schizophrenia and psychotic experiences in our data is counterintuitive and difficult to explain, but it adds further evidence against the hypothesis that the genetic etiology of psychotic experiences is similar to that for schizophrenia. Although sample attrition in our study was not insubstantial, it is hard to envisage how selection bias could explain this finding. Attrition associated with psychotic experiences, family history of psychosis, or with genetic risk for psychosis could lead to estimates of association that are closer to the null, but patterns of attrition that would lead to an apparent protective effect of schizophrenia risk alleles on psychotic experiences are not easily envisaged.

We also examined the association between a genome-wide array of variants and psychotic experiences. No variants showed genome-wide evidence of association, but although this is one of the largest population-based studies of psychotic experiences to date, we had very low statistical power to find such strength of evidence, given the magnitude of genetic effects described for most multifactorial complex outcomes, the number of events in our study, and the threshold required for genome-wide evidence of association.

A number of SNPs showed suggestive evidence (*P* < 5 × 10^−5^) for association with psychotic experiences, but in the context of the number of markers examined, such findings are clearly likely to be chance observations. Although some of these SNPs are within genes for which some evidence of association with schizophrenia in the literature also exists,^11,34^ this is not surprising given the number of genes that have been associated with schizophrenia, as well as the diversity of functional pathways in the brain that are hypothesized to be disrupted in this disorder. The most important finding from this GWAS, however, is not the strength of evidence of specific genes but the absence of evidence that the SNPs, showing strongest evidence of association with psychotic experiences in our study, were enriched for variants associated with schizophrenia in the PGC-SCZ sample.

If causal variants for such experiences could be identified, then studying the effects of these genes on neuroimaging, cognitive, and psychological phenotypes, as well as their impact on development of psychosis throughout the life course, in population-based longitudinal studies, such as the one used for this current study, could be a valuable asset in understanding pathological mechanisms underlying disorders such as schizophrenia. However, that even the present sample is underpowered to detect such alleles points to the enormous challenges faced by population studies.

The thesis that studies of psychotic experiences in the general population may allow us to increase our understanding of the etiology of psychotic disorders relies upon the premise that the presence of such phenomena are an early expression of a pathology that, at its more extreme end, results in schizophrenia. However, the genetic overlap between psychotic experiences in the general population and schizophrenia is likely to be too limited to support the use of such experiences as an intermediate phenotype for schizophrenia.

Although the importance of psychotic experiences in general population samples and their relevance to disorders such as schizophrenia remains to be determined,^14,35^ the presence of such experiences may still impair functioning and cause substantial disability,^7,36^ and understanding their etiology could, therefore, be important in its own right. However, the assumption that it will be easier to understand the underlying mechanisms for such extended phenotypes or endophenotypes than for rarer and more severe disorder, such as schizophrenia, is unlikely to hold true.^37^ The potential advantages of being able to study a phenotype in a population-based sample, where problems of selection bias are likely to be minimized and where genetic effects on the development of psychosis over the life course can be examined, have to be weighed against the disadvantage of studying a phenotype that potentially has a more heterogeneous etiology than schizophrenia.

Our findings, and in particular the magnitude of the effect we observed for the polygenic score compared with that for schizophrenia, indicate that the genetic architecture of psychotic experiences in the general population is not likely to be comparable to that underlying schizophrenia. The implication of this is that while samples may be easier to collect, genetic studies of psychotic experiences are unlikely to aid understanding of the genetic etiology of schizophrenia, or, at the very least, will be substantially underpowered compared to equivalent-sized samples of individuals with this disorder. It is possible that similar evaluation of other phenotypes, such as deficits in specific neurocognitive domains or abnormalities in neuroimaging pathways, may prove to be more informative in addressing this aim.

## Supplementary Material

Supplementary material is available at http://schizophrenia bulletin.oxfordjournals.org.

## Funding

This study was funded by a clinical scientist fellowship awarded to S.Z. by the Welsh Assembly Government and by a Medical Research Council Grant (G0701503). M.J.O. and M.C.O.D. were additionally supported by an MRC Programme Grant (G0800509), MRC Centre Grant (G0801418), and the European Community’s Seventh Framework Programme (HEALTH-F2-2010–241909 [Project EU-GEI]). The UK Medical Research Council, the Wellcome Trust (grant ref.: 092731), and the University of Bristol provide core support for ALSPAC.

## Supplementary Material

Supplementary Data
