# Supplementary material for: A Population-Based Study of Genetic Variation and Psychotic Experiences in Adolescents
Source: Schizophr Bull. 2013 Oct 30;40(6):1254–62. doi: 10.1093/schbul/sbt146 (PMC4193688; doi:10.1093/schbul/sbt146)
Supplement: Supplementary Data [file supp_40_6_1254__index.html]

A Population-Based Study of Genetic Variation and Psychotic Experiences in Adolescents — A Population-Based Study of Genetic Variation and Psychotic Experiences in Adolescents — Supplementary Data 

# A Population-Based Study of Genetic Variation and Psychotic Experiences in Adolescents

## Supplementary Data

Data files

**Files in this Data Supplement:**

- Supplementary Data - Supplementary Data
