# Supplementary material for: A Population-Based Study of Genetic Variation and Psychotic Experiences in Adolescents
Source: Schizophr Bull. 2013 Oct 30;40(6):1254–62. doi: 10.1093/schbul/sbt146 (PMC4193688; doi:10.1093/schbul/sbt146)
Supplement: Supplementary Data [file supp_sbt146_Zammit_Supplementary_Tables_22_07_13.doc]

**Supplementary Table S1:** Association (OR (95% CI)) between Schizophrenia Polygenic Score Quintiles and definite psychotic experiences at age 12 years or age 18 years

|  |  | **P-value cut-off in discovery sample** | | | | | | |
| --- | --- | --- | --- | --- | --- | --- | --- | --- |
|  |  | **0.5** | **0.4** | **0.3** | **0.2** | **0.1** | **0.05** | **0.01** |
| **Quintile of polygenic score** | **1 (lowest)** | 1 (reference) | 1 (reference) | 1 (reference) | 1 (reference) | 1 (reference) | 1 (reference) | 1 (reference) |
|  | **2** | 1.00 (0.72, 1.39) | 0.97 (0.70, 1.34) | 0.85 (0.61, 1.17) | 0.94 (0.68, 1.31) | 1.24 (0.89, 1.71) | 0.95 (0.68, 1.32) | 1.16 (0.83, 0.62) |
|  | **3** | 1.03 (0.74, 1.43) | 0.91 (0.65, 1.27) | 0.82 (0.58, 1.14) | 1.18 (0.86, 1.63) | 1.16 (0.83, 1.62) | 1.02 (0.73, 1.42) | 1.21 (0.87, 1.69) |
|  | **4** | 1.15 (0.83, 1.59) | 1.20 (0.88, 1.64) | 1.30 (0.96, 1.77) | 0.96 (0.69, 1.33) | 1.20 (0.86, 1.67) | 1.22 (0.88, 1.68) | 1.20 (0.86, 1.67) |
|  | **5 (highest)** | 1.23 (0.89, 1.69) | 1.07 (0.77, 1.48) | 1.09 (0.79, 1.50) | 1.34 (0.98, 1.83) | 1.37 (0.99, 1.90) | 1.24 (0.89, 1.71) | 1.30 (0.93, 1.82) |
| **p-value^1^** |  | 0.642 | 0.516 | 0.029 | 0.143 | 0.431 | 0.364 | 0.635 |

^1^Overall p-value (chi-squared test with 4 degrees of freedom)

**Supplementary Table S2:** Association between schizophrenia candidate SNPs and definite psychotic experiences at age 12 or age 18

| **CHR** | **GENE^a^** | **SNP^b^** | **A1^d^** | **A2^d^** | **FREQ(A1)** | **OR^e^** | **LCI** | **UCI** | **P** |
| --- | --- | --- | --- | --- | --- | --- | --- | --- | --- |
| 1 | PTBP2 | rs1591865 | C | G | 0.822 | 1.03 | 0.85 | 1.25 | 0.764 |
| 1 | DISC1 | rs3738401 | A | G | 0.312 | 1.12 | 0.96 | 1.31 | 0.147 |
| 1 | DISC1 | rs6675281 | C | T | 0.865 | 1.03 | 0.83 | 1.27 | 0.806 |
| 1 | DISC1 | rs821616 | A | T | 0.719 | 0.89 | 0.76 | 1.05 | 0.161 |
| **2** | **NRXN1** | **rs3850333** | **A** | **G** | **0.417** | **0.82** | **0.71** | **0.95** | **0.009** |
| 2 | ZNF804A | rs17584522 | C | G | 0.738 | 0.96 | 0.81 | 1.13 | 0.616 |
| 2 | ZNF804A | rs1583048 | C | T | 0.199 | 1.01 | 0.84 | 1.21 | 0.917 |
| **2** | **ERBB4** | **rs4673628** | **A** | **G** | **0.536** | **1.21** | **1.04** | **1.40** | **0.012** |
| 2 | ERBB4 | rs7598440 | C | T | 0.442 | 0.96 | 0.83 | 1.11 | 0.555 |
| 3 | PTPRG | rs349158 | C | T | 0.209 | 1.10 | 0.93 | 1.31 | 0.253 |
| 3 | FXR1 | rs7640601 | C | G | 0.708 | 0.93 | 0.79 | 1.10 | 0.394 |
| 6 | DTNBP1 | rs4715984 | A | G | 0.100 | 1.07 | 0.84 | 1.35 | 0.603 |
| 6 | DTNBP1 | rs3213207 | C | T | 0.116 | 0.93 | 0.74 | 1.17 | 0.549 |
| 6 | DTNBP1 | rs2619538 | A | T | 0.407 | 1.02 | 0.88 | 1.19 | 0.775 |
| 6 | PRSS16 | rs13219354 | C | T | 0.140 | 1.01 | 0.82 | 1.24 | 0.957 |
| 6 | PRSS16 | rs7746199 | C | T | 0.807 | 1.06 | 0.88 | 1.27 | 0.572 |
| 6 | PGBD1 | rs13211507 | C | T | 0.118 | 0.98 | 0.79 | 1.23 | 0.887 |
| 6 | TRIM26 | rs2844776 | C | T | 0.229 | 0.91 | 0.76 | 1.08 | 0.276 |
| 6 | HLA-DQA1 | rs9272219 | G | T | 0.734 | 0.95 | 0.81 | 1.12 | 0.564 |
| 6 | HLA-DQA1 | rs9272105^c^ | A | G | 0.479 | 0.96 | 0.83 | 1.10 | 0.556 |
| 6 | QKI | rs9295224 | C | G | 0.314 | 1.02 | 0.87 | 1.19 | 0.850 |
| **7** | **GRM3** | **rs6465084**^c^ | **G** | **A** | **0.244** | **1.19** | **1.00** | **1.42** | **0.048** |
| 7 | MAD1L1 | rs10226475 | A | G | 0.585 | 0.93 | 0.80 | 1.08 | 0.330 |
| 7 | MAGI2 | rs4296979 | C | T | 0.122 | 1.08 | 0.87 | 1.34 | 0.507 |
| **7** | **MAGI2** | **rs1207881** | **C** | **T** | **0.696** | **1.19** | **1.01** | **1.40** | **0.038** |
| **7** | **MAGI2** | **rs6951046**^c^ | **T** | **C** | **0.421** | **0.84** | **0.72** | **0.97** | **0.017** |
| 7 | PTPRZ1 | rs2024249 | A | G | 0.459 | 0.97 | 0.84 | 1.12 | 0.687 |
| 7 | PTPRZ1 | rs6466808 | A | G | 0.381 | 0.90 | 0.78 | 1.05 | 0.200 |
| 7 | PTPRZ1 | rs1147498 | A | C | 0.811 | 1.12 | 0.92 | 1.36 | 0.261 |
| 8 | CSMD1 | rs10503256 | A | G | 0.363 | 1.04 | 0.90 | 1.22 | 0.566 |
| 8 | MMP16 | rs7004633 | A | G | 0.838 | 1.19 | 0.96 | 1.47 | 0.107 |
| 8 | NRG1 | nrg221132^c^ | A | G | 0.113 | 0.98 | 0.77 | 1.24 | 0.838 |
| 8 | NRG1 | nrg221533^c^ | C | T | 0.362 | 1.06 | 0.90 | 1.24 | 0.497 |
| 8 | NRG1 | nrg241930^c^ | T | G | 0.357 | 0.95 | 0.81 | 1.11 | 0.501 |
| 8 | NRG1 | nrg243177^c^ | T | C | 0.400 | 1.09 | 0.93 | 1.27 | 0.294 |
| 10 | ANK3 | rs1938526 | A | G | 0.939 | 0.88 | 0.66 | 1.18 | 0.386 |
| 11 | KCNA4 | rs1602565 | C | T | 0.118 | 0.94 | 0.75 | 1.17 | 0.569 |
| 11 | DRD2 | rs6277 | A | G | 0.549 | 1.04 | 0.90 | 1.21 | 0.555 |
| 11 | NRGN | rs12807809 | C | T | 0.173 | 1.07 | 0.89 | 1.29 | 0.478 |
| 11 | STT3A/EI24 | rs548181 | A | G | 0.126 | 0.92 | 0.74 | 1.15 | 0.461 |
| 12 | CACNA1C | rs1006737 | A | G | 0.338 | 0.97 | 0.83 | 1.13 | 0.658 |
| 12 | NOS1 | rs6490121 | A | G | 0.660 | 0.88 | 0.75 | 1.02 | 0.090 |
| 13 | G72 | rs2391191 | A | G | 0.381 | 0.98 | 0.84 | 1.14 | 0.791 |
| 14 | PSMA6 | rs12436216 | A | G | 0.426 | 0.98 | 0.85 | 1.13 | 0.781 |
| 15 | CHRNA5A3B4 | rs578776 | A | G | 0.277 | 1.02 | 0.86 | 1.19 | 0.858 |
| 16 | FLJ11151 | rs7192086 | A | T | 0.745 | 0.89 | 0.75 | 1.06 | 0.195 |
| 16 | RPGRIP1L | rs9922369 | A | G | 0.029 | 0.89 | 0.57 | 1.40 | 0.613 |
| 17 | CNP | rs2070106 | A | G | 0.332 | 1.03 | 0.87 | 1.20 | 0.756 |
| 17 | PMP22 | PMP22_t118m^c^ | T | C | 0.005 | 0.84 | 0.29 | 2.41 | 0.748 |
| 18 | TCF4 | rs17512836^c^ | C | T | 0.025 | 0.96 | 0.62 | 1.50 | 0.859 |
| 18 | CCDC68 | rs12966547 | A | G | 0.398 | 1.13 | 0.97 | 1.31 | 0.109 |
| 18 | TCF4 | rs9960767 | A | C | 0.948 | 1.03 | 0.73 | 1.44 | 0.881 |
| 21 | OLIG2 | rs1005573 | C | T | 0.324 | 0.98 | 0.84 | 1.15 | 0.792 |
| 21 | OLIG2 | rs1059004^c^ | C | A | 0.441 | 0.95 | 0.82 | 1.10 | 0.491 |
| 22 | TBX1 | rs2301558 | C | T | 0.769 | 1.04 | 0.87 | 1.23 | 0.695 |
| **22** | **GNB1L** | **rs2269726** | **C** | **T** | **0.342** | **0.84** | **0.72** | **0.98** | **0.031** |
| 22 | COMT | rs165599^c^ | G | A | 0.295 | 0.97 | 0.82 | 1.15 | 0.738 |
| **22** | **COMT** | **rs2097603**^c^ | **G** | **A** | **0.433** | **1.17** | **1.00** | **1.36** | **0.050^f^** |
| 22 | COMT | rs737865 | A | G | 0.707 | 1.10 | 0.93 | 1.30 | 0.287 |
| 22 | COMT | rs4818 | C | G | 0.595 | 0.93 | 0.81 | 1.08 | 0.337 |
| 22 | COMT | rs4680 | A | G | 0.519 | 0.97 | 0.84 | 1.12 | 0.642 |

^a^ Or closest gene if intergenic; ^b^All SNPs r^2^<0.8; ^c^ Not in array, genotyped separately; ^d^ Risk allele for schizophrenia underlined if evidence (p<5x10^-4^) of association in PGC-SCZ; ^e^ Per Allele 1; ^f^ As a further test that alterations in COMT enzyme activity may be associated with psychosis we examined whether psychotic experiences were associated with 3-marker haplotypes (rs6269, rs4818 and rs4680; rs4633 omitted as r^2^ of 1 with rs4680) that more accurately reflect protein activity and enzyme function than rs4680 individually (Nackl*ey et a*l., 2006), but there was no evidence that either the high-activity or low activity haplotypes were associated with our outcome (p = 0.884 and p = 0.583 respectively).

**Supplementary Table S3:** Association between Schizophrenia Polygenic Score (per SD) and suspected or definite PEs at age 12 or age 18

| **P-value cut-off in discovery sample** | **OR** | **LCI** | **UCI** | **P** | **Same direction as discovery sample** |
| --- | --- | --- | --- | --- | --- |
| 0.5 | 1.05 | 0.97 | 1.13 | 0.254 | + |
| 0.4 | 1.04 | 0.97 | 1.12 | 0.272 | + |
| 0.3 | 1.05 | 0.98 | 1.14 | 0.172 | + |
| 0.2 | 1.06 | 0.98 | 1.14 | 0.163 | + |
| 0.1 | 1.06 | 0.98 | 1.14 | 0.139 | + |
| 0.05 | 1.07 | 0.99 | 1.15 | 0.091 | + |
| 0.01 | 1.05 | 0.97 | 1.13 | 0.219 | + |

*No evidence of non-linearity (including quadratic terms)

**Supplementary Table S4:** Association between schizophrenia GWS SNPs and suspected or definite PEs at age 12 or age 18

| **Ch** | **Gene** | **SNP** | **Allele^a^** | **OR^b^** | **L95** | **U95** | **P** | **Same as SZ^c^** |
| --- | --- | --- | --- | --- | --- | --- | --- | --- |
| 1 | MIR137 | rs1625579 | G,T | 1.09 | 0.95 | 1.24 | 0.237 | - |
| 1 | VRK2 (IG) | rs2312147 | **C**,T | 1.00 | 0.90 | 1.12 | 0.990 |  |
| 2 | ZNF804A | rs1344706 | **A**,C | 0.98 | 0.88 | 1.09 | 0.735 | - |
| 2 | PCGEM1 (IG) | rs17662626 | A,G | 0.82 | 0.68 | 0.99 | 0.036 | - |
| 3 | ITIH3/4 | rs2239547 | C,**T** | 1.07 | 0.95 | 1.21 | 0.264 | - |
| 6 | PRSS16 | rs6932590 | C,**T** | 1.00 | 0.88 | 1.13 | 0.999 |  |
| 6 | TRIM26 | rs2021722 | **C**,T | 1.07 | 0.94 | 1.21 | 0.293 | + |
| 6 | NOTCH4 | rs3131296 | **C**,T | 1.00 | 0.86 | 1.15 | 0.949 |  |
| 8 | CSMD1 | rs10503253 | **A**,C | 1.03 | 0.90 | 1.17 | 0.717 | + |
| 8 | MMP16 (IG) | rs7004633 | A,**G** | 1.07 | 0.92 | 1.24 | 0.387 | - |
| 10 | CNNM2 | rs7914558 | A,**G** | 0.98 | 0.88 | 1.10 | 0.764 | + |
| 10 | NT5C2 | rs11191580 | C,T | 1.07 | 0.88 | 1.31 | 0.497 | - |
| 11 | NRGN | rs12807809 | C,**T** | 0.92 | 0.80 | 1.06 | 0.249 | + |
| 11 | AMBRA1 | rs11819869 | **C,T** | 1.02 | 0.88 | 1.17 | 0.830 |  |
| 12 | CACNA1C | rs4765905 | **C**,G | 0.99 | 0.89 | 1.11 | 0.862 | - |
| 18 | CCDC68 (IG) | rs12966547 | A,**G** | 1.07 | 0.96 | 1.20 | 0.194 | - |
| 18 | TCF4 | rs9960767 | A,**C** | 0.96 | 0.76 | 1.23 | 0.772 | + |
|  |  |  |  |  |  |  |  |  |
| Risk score (17 GWS hits) | | |  | 0.98 | 0.95 | 1.01 | 0.221 |  |

**Supplementary Table S5: GWAS of PEs: PE1218 suspected/definite vs none**

|  |  |  | **ALSPAC** | | | | | | | **PGC-SCZ** |
| --- | --- | --- | --- | --- | --- | --- | --- | --- | --- | --- |
| CHR | GENE | SNP | ALLELE | FREQ1 | RSQR | OR | LCI | UCI | P | P |
| 1 | null | rs2880538 | C,T | 0.0939 | 0.9989 | 1.51 | 1.27 | 1.80 | 3.52E-06 | 0.383 |
| 2 | null | rs4851262 | A,C | 0.7657 | 0.9987 | 1.32 | 1.16 | 1.50 | 3.48E-05 | 0.450^1^ |
| 3 | GPR149 | rs2872337 | A,C | 0.1574 | 0.9992 | 1.37 | 1.19 | 1.57 | 1.30E-05 | 0.316 |
| 3 | null | rs9874793 | A,G | 0.4774 | 0.9649 | 0.80 | 0.71 | 0.89 | 4.54E-05 | 0.520 |
| 5 | null | rs4700481 | A,G | 0.5908 | 0.9268 | 0.79 | 0.71 | 0.89 | 4.95E-05 | 0.508 |
| 5 | null | rs6453460 | A,T | 0.1714 | 0.9088 | 1.39 | 1.20 | 1.61 | 1.10E-05 | 0.507 |
| 5 | DOCK2/LOC100131897 | rs166354 | A,G | 0.6617 | 0.9921 | 0.79 | 0.70 | 0.88 | 3.87E-05 | 0.188 |
| 5 | null | rs1317022 | C,T | 0.6501 | 0.9999 | 0.78 | 0.70 | 0.87 | 1.35E-05 | 0.767 |
| 6 | null | rs4445052 | A,G | 0.1369 | 0.9768 | 1.37 | 1.18 | 1.59 | 4.71E-05 | 0.341 |
| 6 | COL11A2 | rs2855458 | C,G | 0.0101 | 0.9386 | 2.70 | 1.69 | 4.33 | 3.28E-05 | 0.382 |
| 6 | SASH1 | rs12204885 | C,G | 0.9642 | 0.9658 | 0.58 | 0.44 | 0.75 | 4.29E-05 | 0.426 |
| 7 | PDE1C | rs17335283 | A,C | 0.9655 | 0.9938 | 0.54 | 0.41 | 0.70 | 7.71E-06 | 0.506 |
| 7 | MAGI2 | rs10254500 | C,T | 0.2085 | 0.9983 | 1.33 | 1.17 | 1.51 | 1.40E-05 | 0.936 |
| 7 | null | rs17520243 | A,G | 0.7723 | 0.4133 | 1.58 | 1.28 | 1.95 | 1.75E-05 | 0.951 |
| 7 | MKRN1 | rs4431522 | C,G | 0.1194 | 0.7682 | 1.48 | 1.24 | 1.77 | 1.90E-05 | 0.504 |
| 8 | null | rs2840450 | A,T | 0.4924 | 0.9072 | 0.79 | 0.71 | 0.89 | 4.84E-05 | 0.991 |
| **9** | **null** | **rs10511602** | **C,T** | **0.1343** | **0.996** | **0.70** | **0.59** | **0.83** | **2.80E-05** | **0.009^2^** |
| 9 | null | rs1934735 | C,T | 0.7806 | 0.997 | 1.34 | 1.16 | 1.54 | 4.34E-05 | 0.895 |
| 9 | C9orf98 | rs2809255 | A,G | 0.0798 | 0.9995 | 1.53 | 1.28 | 1.84 | 4.62E-06 | 0.364 |
| 10 | null | rs7090259 | C,T | 0.2744 | 0.819 | 1.40 | 1.23 | 1.59 | 2.98E-07 | 0.291 |
| 10 | C10orf11 | rs12245471 | A,T | 0.6626 | 0.4201 | 1.46 | 1.22 | 1.75 | 3.79E-05 | 0.733 |
| 11 | null | rs10769111 | G,T | 0.219 | 0.4233 | 1.57 | 1.29 | 1.90 | 4.95E-06 | 0.486 |
| 11 | STARD10 | rs11235585 | A,G | 0.0731 | 0.9728 | 1.52 | 1.25 | 1.85 | 2.13E-05 | 0.071 |
| 11 | null | rs7482532 | C,T | 0.6732 | 0.9982 | 0.78 | 0.70 | 0.87 | 1.56E-05 | 0.614 |
| 12 | ITPR2 | rs11048510 | C,G | 0.2496 | 0.9831 | 1.30 | 1.15 | 1.47 | 2.00E-05 | 0.752 |
| 12 | null | rs1879390 | A,C | 0.1164 | 0.966 | 1.44 | 1.22 | 1.69 | 1.20E-05 | 0.837 |
| 13 | null | rs7987907 | C,T | 0.5803 | 0.9987 | 0.78 | 0.70 | 0.87 | 1.53E-05 | 0.254 |
| 15 | null | rs7495326 | A,G | 0.2006 | 0.9281 | 1.34 | 1.17 | 1.54 | 1.66E-05 | 0.762 |
| 16 | ZNF423 | rs8060387 | C,G | 0.362 | 0.9156 | 1.28 | 1.14 | 1.44 | 2.97E-05 | 0.238 |
| 16 | null | rs12448442 | A,G | 0.8305 | 0.9867 | 0.74 | 0.64 | 0.85 | 2.59E-05 | 0.091 |
| 18 | null | rs7232121 | C,G | 0.5627 | 0.9364 | 1.28 | 1.14 | 1.43 | 2.90E-05 | 0.078 |
| 18 | null | rs4450496 | G,T | 0.0482 | 0.8445 | 1.72 | 1.34 | 2.22 | 2.37E-05 | 0.550 |
| 19 | MEF2B | rs11671119 | C,T | 0.9781 | 0.8288 | 0.45 | 0.31 | 0.64 | 1.33E-05 | 0.701 |
| 21 | null | rs13048007 | C,T | 0.4458 | 0.9979 | 1.25 | 1.13 | 1.40 | 3.87E-05 | 0.416 |
| 22 | KIAA1671 | rs5760863 | C,T | 0.7099 | 0.9691 | 1.31 | 1.16 | 1.49 | 1.38E-05 | 0.058 |
| 23 | null | rs5924678 | G,C | 0.7118 | 0.7098 | 0.78 | 0.70 | 0.88 | 2.40E-05 | NSM^2^ |

^1^SNP not in PGC-SCZ, p-value is ISC-SGENE-MGS meta-analysis; ^2^same risk allele, p = 0.297 for binomial test for 1 SNP at p<0.01 out of 35 SNPs; ^3^ NSM = no shared marker in PGC-SCZ or ISC-SGENE-MGS meta-analysis
